# Urinary exosomal expression of activator of G protein signaling 3 in polycystic kidney disease

**DOI:** 10.1186/s13104-018-3467-6

**Published:** 2018-06-07

**Authors:** Krishna C. Keri, Kevin R. Regner, Aaron T. Dall, Frank Park

**Affiliations:** 1 Department of Medicine, Division of Nephrology, Medical College of Wisconsin, 8701 Watertown Plank Road, Milwaukee, WI 53226 USA; 2 Department of Pharmaceutical Sciences, University of Tennessee Health Science Center College of Pharmacy, 881 Madison Ave, Rm 442, Memphis, TN 38163 USA

**Keywords:** Polycystic kidney disease, Urine exosomes, Kidney, Western blot analysis, Biomarker

## Abstract

**Objective:**

PKD is a genetic disease that is characterized by abnormally proliferative epithelial cells in the kidney and liver. Urinary exosomes have been previously examined as a source of unique proteins that may be used to diagnose and monitor the progression of PKD. Previous studies by our group have shown that AGS3, which is a receptor-independent regulator G-proteins, was markedly upregulated in RTECs during kidney injury including PKD. In this study, our goal was to determine whether AGS3 could be measured in exosomes using animals and humans with PKD.

**Results:**

In our study, urinary exosomes were isolated from PCK rats and the control Sprague–Dawley (SD) rats. AGS3 expression was significantly increased (P < 0.05) in PKD versus SD rats at 16 weeks of age. This increase was detectable in a time-dependent manner from 8 weeks of age and peaked at ~ 16–20 weeks (length of study). Similarly, in exosomes from human urine samples with PKD, AGS3 expression was significantly increased (P < 0.05) compared to healthy human controls where AGS3 was largely undetectable. In conclusion, the detection of AGS3 in urinary exosomes may be a novel biomarker for PKD, and provide new insight into the biology of tubular epithelial cell function during cystic disease progression.

## Introduction

Polycystic kidney disease in humans is due to mutations in PKD1, PKD2 or PKHD1 genes. PKD is characterized by the ongoing accumulation of fluid in thin walled cysts originating from renal tubular epithelial cells (RTECs), which leads to renal parenchyma damage and non-cystic renal tissue fibrosis [1].

Currently, there are no effective treatments for PKD, due to the complex function of the polycystin proteins and the unpredictable time course over which cyst burden causes renal failure. Surrogate markers are being developed to aid in prognosticating cyst number and size with a loss of renal function. In the Consortium for Radiologic Imaging Studies of Polycystic Kidney Disease (CRISP) study [2], magnetic resonance imaging (MRI) was used to estimate changes in cyst volume and kidney size over time periods as short as 6 months [3]. However, MRI is a costly and resource intensive approach to monitoring progression of PKD. An alternate and cost-effective approach would be to identify urinary biomarkers to monitor PKD progression.

Human urine contains an array of proteins, some of which are differentially expressed in the setting of kidney disease [4]. Urinary proteins can either be soluble or associated with shed tubular epithelial cells or cellular microvesicles. Exosomes are 40–100 nm vesicles that originate as internal microvesicles in multivesicular bodies (MVB) from RTECs facing the urinary space and are excreted into the urine [5–7]. Proteomic analysis of exosomes provides insight into protein expression patterns within RTECs [5–7]. In PKD, a prior proteomic analysis found that exosomes contained 376 novel proteins not previously found in human urine [8].

Here, we focused upon a protein, Activator of G-protein signaling 3 (AGS3), which has not been previously identified in urine, but has been shown to be markedly increased in PKD kidneys [9–11]. AGS3 has biological functions relevant to the pathogenesis of PKD, including regulation of mitotic-spindle orientation, adenylyl-cyclase activity, polycystin ion channel activity, and programmed cell death [9, 12–16]. Moreover, earlier studies demonstrated that several AGS3 interacting proteins, including Gα subunits, were present in urinary exosomes [8]. In this study, we aimed to determine whether AGS3 expression was upregulated in urinary exosomes obtained from rats and humans with PKD.

## Main text

### Methods

#### Animals

Polycystic kidney disease (PCK) rats were obtained from a breeding colony maintained at the Medical College of Wisconsin. The PCK rat was originally identified with a spontaneous 2 base pair deletion in the *PKHD1* gene in a strain of the Sprague–Dawley rat at Charles River in Japan [17]. The PCK rat has been well described as a rodent model of PKD, which reproducibly develops renal and hepatic cysts with a concomitant gradual decline in kidney function [17]. Male Sprague–Dawley (SD) rats were obtained from Taconic Farms (Germantown, NY). Rats were allowed ad libitum access to food and water during the study. All protocols used in this study were approved by the Medical College of Wisconsin Institutional Animal Care and Use Committee.

#### Assessment of renal injury

Subgroups of SD and PCK rats were euthanized at 8, 16, 24, and 26 weeks of age. Prior to euthanasia, blood was harvested by cardiac puncture and plasma was isolated to measure creatinine [18, 19]. Kidneys were formalin fixed, paraffin-embedded, sectioned (4 μm), and stained with hematoxylin & eosin (H&E). Representative images were acquired using a Nikon 55i light microscope and Nis-Elements image analysis software (version 3.03, Nikon Instruments Inc., Melville, NY).

#### Human subjects

Healthy control subjects and subjects with autosomal dominant PKD were identified at the Medical College of Wisconsin clinics. This study was approved by the Institutional Review Board at the Medical College of Wisconsin prior to the initiation of this study. Subjects were contacted by a member of the study team at the time of the clinic visit to inquire about their willingness to participate if they met the eligibility criteria: (1) 18 years of age and older; and (2) able to provide written informed consent. Subjects were considered as healthy controls if they or their family members had no history of kidney disease. Subjects were classified into the PKD group if they carried a clinical diagnosis of autosomal dominant PKD made by an attending nephrologist. The exclusion criteria were: (1) estimated glomerular filtration rate (eGFR) of < 20 mL/min; (2) history of prior kidney transplant or other solid organ transplant; (3) history of acute kidney injury; or (4) unable to provide written informed consent.

Following the informed consent process, clinical and demographic data (age, gender, serum creatinine, eGFR, and spot urine protein/creatinine) were collected by review of medical records. Spot urine samples were collected from healthy control subjects (n = 7) and patients with PKD (n = 7).

#### Isolation of urinary exosomes

SD (n = 8) and PCK (n = 6) rats were placed in metabolic cages for 24 h at 8, 12, 16, and 20 weeks of age. Exosomes were isolated from the 24-h urine samples using a modified method previously described [6, 7]. From the human subjects, a clean-catch midstream urine sample was obtained. For both the rat and human samples, a tablet of Complete Mini-protease Inhibitor Cocktail (Roche, Indianapolis, IN) was added to each specimen at the time of collection. Urine samples were then placed on ice and immediately prepared for centrifugation by transferring to ultracentrifuge tubes. Phosphate buffered saline was added to each sample to yield a final volume of 34 mL/sample. The urine was differentially centrifuged at 15,000 and 150,000×*g* for 60 min at 4 °C. The supernatant was discarded at each step, and the high-speed spin was repeated to increase the yield of exosomes. The final pellet was resuspended in 250 µL of 1× RIPA buffer. Urinary-exosomal protein yield for each specimen was measured with the DC Protein Assay Kit II (Biorad, Hercules, CA). The presence of urinary exosomes in the high-speed urine pellets was confirmed by electron microscopy (Electron Microscopy Core at the Medical College of Wisconsin).

#### AGS3 protein detection in rat kidney tissue and human and rat urinary exosomes

SD and PCK rat kidney lysates at 8, 16, and 24 weeks of age, and human and rat urine exosome protein lysates were isolated using 1X RIPA buffer containing protease (Roche) and phosphatase inhibitors (Pierce, Rockford, IL). AGS3 protein detection was determined using standard Western blot techniques [9, 11, 20]. Mouse anti-β-actin (1:4000; cat #A5441, Sigma, St. Louis, MO) was used as a loading control. Rat brain lysates were used as a positive control, since AGS3 is enriched in brain tissue [21]. Band intensities were quantified with NIH Image J software [9, 11, 20], and the densitometry values were calculated using a method modified from Esteva-Font et al. [22].

#### Statistical analysis

The significance of differences between groups was assessed by the Mann–Whitney test or Fisher’s exact test. P < 0.05 was considered statistically significant. Statistical analyses were performed using Prism 4.0 software (GraphPad Software Inc., San Diego CA). Unless specified otherwise, data are presented as median (range). For clarity of graphical presentation, normalized densitometry values were log2 transformed.

### Results

#### Animal study

**Fig. 1:**
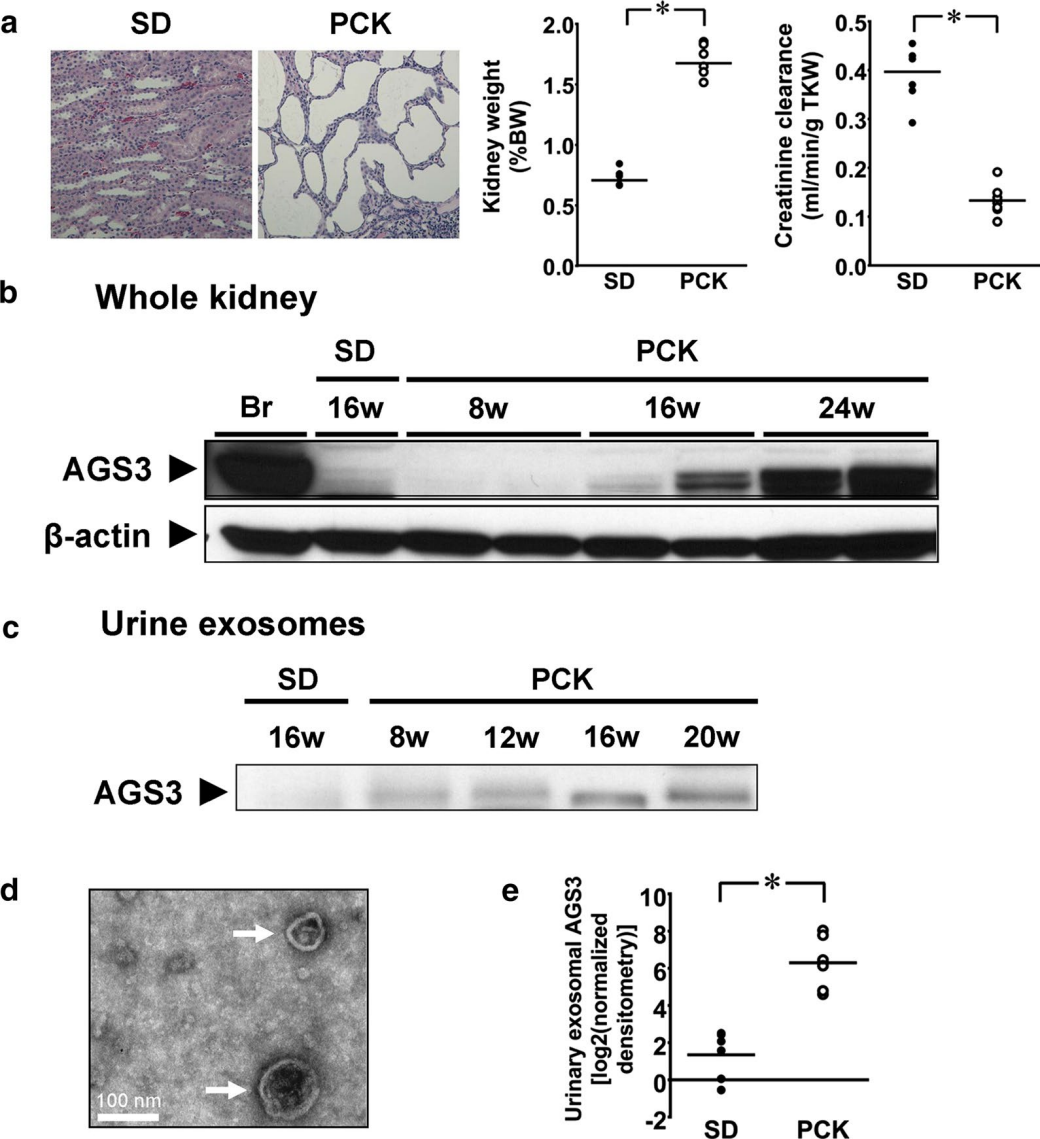
Detection of AGS3 in rat whole kidney and urinary exosomes. **a** Representative photomicrographs SD and PCK rat kidneys at 24 weeks are presented (original magnification, ×10). Circles indicate individual values. Line indicates group median. *P < 0.05. BW: body weight. TKW: total kidney weight. **b** Representative Western blot demonstrating AGS3 expression in SD and PCK rat kidneys at 8, 16, and 24 weeks of age. Rat brain was used as a positive control for AGS3. β-actin was used as a loading control. **c** Time course change in AGS3 protein in urine exosomes. SD and PCK rats were placed in metabolic cages to collect urine for 24 h at 8, 12, 16 and 20 weeks of age, and a Western blot of AGS3 using the proteins isolated from rat urinary exosomes is shown for SD rats (16 weeks) and PCK rats (8, 12, 16 and 20 weeks). **d**, **e** Urine exosome collection in rats and measurement of AGS3 protein by Western blot analysis. SD (n = 8) and PCK (n = 6) rats were placed in metabolic cages to collect urine for 24 h at 16 weeks of age. Urine samples were then centrifuged to isolate urinary exosomes. **d** The presence of urinary exosomes (arrows) in rat urine was confirmed by electron microscopy. Scale bar = 100 nm. **e** AGS3 protein expression in urinary exosomes from 16-week-old SD and PCK rat was measured by densitometry. Circles represent individual log2 transformed values. Line indicates group mean. *P < 0.05

By 24 weeks, PCK rat kidneys demonstrated numerous cysts and a significant increase in the kidney-to-total body weight ratio and an associated decrease in creatinine clearance in comparison to SD control rats (Fig. 1a). As shown in Fig. 1b, AGS3 expression in the whole kidney lysates temporally increased from 8 to 24 weeks of age in the PCK rats, which is consistent with a previous study associating AGS3 expression with cyst progression [11].

The median (range) 24-h urine volume in SD rats was 15.5 (8–38) mL and did not significantly differ from the volume in PCK rats [23.0 (12–28) mL, P = 0.3]. The presence of 40–80 nm exosomes in the rat urine samples was confirmed by electron microscopy (arrows Fig. 1d). AGS3 immunoblotting was performed on urinary exosomes derived from SD and PCK rats between 8 and 20 weeks of age (Fig. 1c, e). Minimal AGS3 expression was detected in the exosomes isolated from the SD rats (Fig. 1c), but there was a temporal increase in the AGS3 expression from urinary exosomes similar to the kidney tissue (Fig. 1b**)**. Urinary AGS3 expression was significantly increased (P = 0.002) in PCK exosomes in comparison to control SD exosomes at 16 weeks of age (Fig. 1e**)**, and AGS3 could be detected as early as 8 weeks in the PCK rat urine.

#### Human study

Spot urine samples were collected from healthy control subjects (n = 7) and patients with PKD (n = 7). The characteristics of the study subjects are presented in Table 1. Age and gender did not significantly differ between the healthy control group and the PKD group. eGFR was significantly lower (P < 0.05) in the PKD group.

**Table 1:** Subject characteristics

	Control (n = 7)	PKD (n = 7)	P
Age (years)	41 (34–68)	48 (24–56)	0.9
Females	3 (43)	4 (57)	0.9
Serum creatinine (mg/dL)	1.04 (0.89–1.15)	1.09 (0.89–1.65)	0.8
eGFR (mL/min/1.73 m^2^)	80 (60–101)	60 (32–60)	0.03

Data are presented as median (range) or number (%)

*eGFR* estimated glomerular filtration rate

**Fig. 2:**
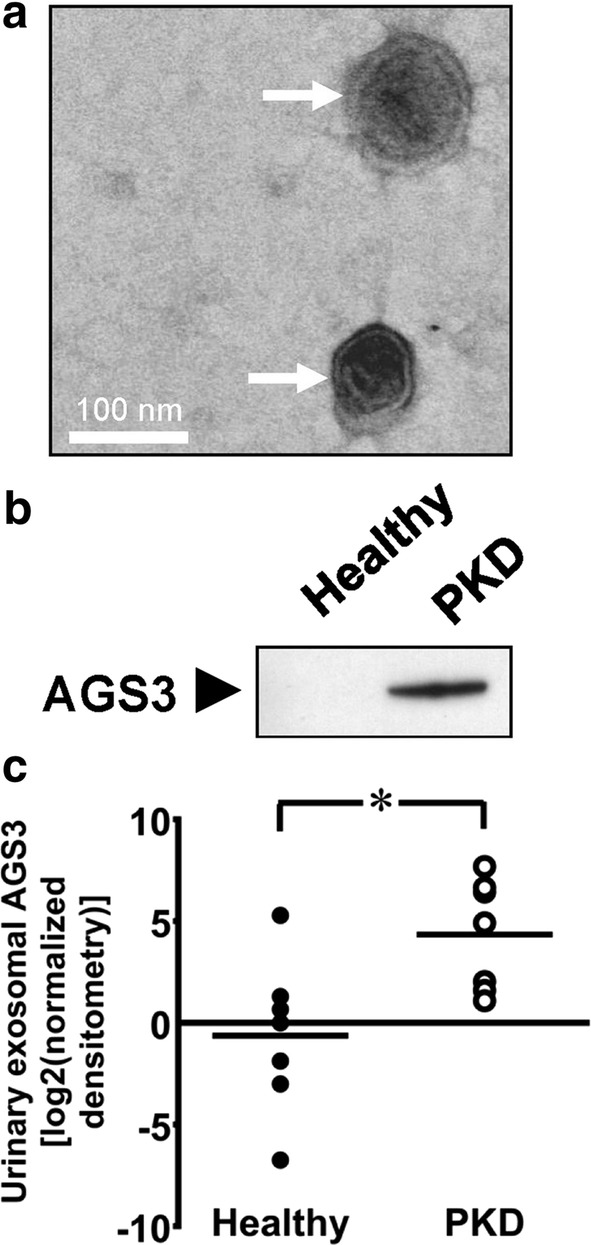
Detection of AGS3 in human urinary exosomes. Spot urine samples were collected from human healthy human subjects and patients with PKD (n = 7/group). Urine samples were then centrifuged to isolate urinary exosomes. **a** The presence of urinary exosomes (arrows) in human urine was confirmed by electron microscopy. Scale bar = 100 nm. **b** Representative AGS3 expression in healthy controls and subjects with PKD or CKD was detected by Western blot analysis. **c** AGS3 protein expression in human urinary exosomes was measured by densitometry. Circles represent individual log2 transformed values. Line indicates group mean. *P < 0.05

The presence of 40-80 nm exosomes in the high-speed pellets was confirmed by electron microscopy (arrows, Fig. 2a). Robust expression of AGS3 was detected by immunoblot analysis in urinary exosomes isolated from the PKD patients, but not in exosomes from healthy control subjects (Fig. 2b). Quantitatively, AGS3 band intensities demonstrated a significant increase (P = 0.01) in the AGS3 expression levels in urinary exosomes from subjects with PKD in comparison to healthy controls (Fig. 2c).

### Discussion

An ideal urinary biomarker to monitor PKD progression would have the following characteristics: (1) detectable in spot urine samples; (2) differentially expressed in healthy and cystic kidneys; (3) changes in biomarker expression should precede anatomic and functional alterations in the kidney; and (4) the biomarker should be biologically related to the pathogenesis of PKD [23].

In the present study, AGS3 was chosen as a candidate biomarker of PKD progression for several reasons. First, AGS3 is expressed at low levels in normal kidneys, but is markedly upregulated in PKD [9, 11]. Second, knockout of AGS3 in mice accelerated cystic disease progression suggesting a role to slow PKD pathogenesis [9]. Lastly, a number of proteins known to interact with AGS3 have been previously identified in urinary exosomes [8]. Here, we demonstrate significantly increased expression of AGS3 in urinary exosomes from rats and humans with PKD compared to their healthy counterparts.

The cellular pathways involved in the secretion of exosomes into the urine from the tubular epithelial cells remains largely unknown. Recent studies have suggested that AGS3 functions as a beneficial repair protein in tubular epithelial cells [9, 20], and could facilitate trafficking of select proteins to the plasma membrane [24]. This mode of action would involve the restoration of the normal architecture of the plasma membrane by returning some of the mis localized protein constituents necessary to maintain normal tubular epithelial cell function. Since there is evidence that exosomes deliver their contents (e.g. protein, mRNA) from their cell of origin to various acceptor cells [25], the potential role for AGS3 to act as a trafficking protein by the exosomes to “acceptor” cells enabling the transfer of distinct exosomal proteins or expressed transcripts to another tubular segments is an intriguing scenario.

From our study, the potential utility of AGS3 as a urinary biomarker of PKD progression appears to have some merit. Renal failure occurs slowly in PKD and limits the usefulness of functional markers of renal disease in assessing the progression of PKD. In contrast, recent studies using serial MRI measurements of kidney volume have documented disease progression in time periods as short as 6–12 months [3]. Besides the cost issue, it is known that the cyst progression is accelerated in early life when MRI may have insufficient resolution [26]. This study demonstrates for the first time that urinary-exosomal AGS3 was differentially expressed in healthy subjects and patients with PKD. In rats, AGS3 appeared to be temporally associated with cystic disease progression in a rodent model of PKD and was detectable in the PKD urinary exosomes at a time-point earlier than it was first detectable in the kidney. This is consistent with the findings of a study by Pisitkun et al. [6]. which found that many proteins are enriched in exosomes in comparison to kidney tissue homogenates. Taken together, these observations suggest that AGS3 may have the potential to provide a non-invasive method to monitor progression of PKD.

## Limitations

The main limitation in this study is the variability in AGS3 expression for the PKD patient group, which is likely due to the varying state of cystic disease. A longitudinal study, including patients at various stages of disease, will be required to determine whether urinary-exosomal AGS3 expression correlates with disease severity and progression in humans. However, cystic disease progression is dependent upon both size and number of the cysts. Therefore, one challenge in translating our findings to the assessment of PKD progression is the possibility that AGS3 protein levels differ between individual cysts of varying sizes. Measurement of these changes may be necessary to understand the potential utility of AGS3 as a prognostic or diagnostic biomarker for PKD.

## Abbreviations

ADPKD: autosomal dominant polycystic kidney disease
RTECs: renal tubular epithelial cells
CRIPS: consortium for radiologic imaging studies of polycystic kidney disease
MRI: magnetic resonance imaging
AGS3: activator of G protein signaling 3
Br: brain
BW: body weight
CKD: chronic kidney disease
eGFR: estimated glomerular filtration rate
GPCRs: G protein-coupled receptors
H&E: hematoxylin & eosin
µL: microliter
Min: minute
mL: milliliter
MVB: multivesicular bodies
nm: nanometer
PKD: polycystic kidney disease
SD: Sprague–Dawley
TKD: total kidney weight
